# A simple methodology to assess endolysosomal protease activity involved in antigen processing in human primary cells

**DOI:** 10.1186/1471-2121-14-35

**Published:** 2013-08-09

**Authors:** Archana Vaithilingam, Nicole Y Lai, Ellen Duong, Julie Boucau, Yang Xu, Mariko Shimada, Malini Gandhi, Sylvie Le Gall

**Affiliations:** 1 Ragon Institute of MGH, MIT and Harvard, 400 Technology Square, Cambridge, MA 02139, USA; 2 Harvard Medical School, Boston, MA, USA

**Keywords:** Endolysosome, Antigen processing, Proteases, Cathepsins, Protein degradation, Primary cells, Mass spectrometry, T cell epitope production, MHC, HIV

## Abstract

**Background:**

Endolysosomes play a key role in maintaining the homeostasis of the cell. They are made of a complex set of proteins that degrade lipids, proteins and sugars. Studies involving endolysosome contribution to cellular functions such as MHC class I and II epitope production have used recombinant endolysosomal proteins, knockout mice that lack one of the enzymes or purified organelles from human tissue. Each of these approaches has some caveats in analyzing endolysosomal enzyme functions.

**Results:**

In this study, we have developed a simple methodology to assess endolysosomal protease activity. By varying the pH in crude lysate from human peripheral blood mononuclear cells (PBMCs), we documented increased endolysosomal cathepsin activity in acidic conditions. Using this new method, we showed that the degradation of HIV peptides in low pH extracts analyzed by mass spectrometry followed similar kinetics and degradation patterns as those performed with purified endolysosomes.

**Conclusion:**

By using crude lysate in the place of purified organelles this method will be a quick and useful tool to assess endolysosomal protease activities in primary cells of limited availability. This quick method will especially be useful to screen peptide susceptibility to degradation in endolysosomal compartments for antigen processing studies, following which detailed analysis using purified organelles may be used to study specific peptides.

## Background

Endolysosomes are cellular organelles that play a key role in protein turnover and homeostasis of the cell. They are made up of a complex set of enzymes that break down proteins, lipids and sugars that are all essential for normal functioning of the cell [1]. A genetic mutation in some of these endolysosomal proteins can lead to disorders such as Gaucher’s disease, Niemann pick’s disease, and Fabry disease [2-5]. Endolysosomes also play a key role in antigen presentation by processing large proteins into epitopes that can be presented by MHC Class I and Class II molecules [6-8]. Therefore it is important to develop methodologies suitable to study specific endolysosomal functions in relevant cells and tissues.

Several methods have been used to study their functions in the cell. The approaches taken to study endolysosome contribution to protein degradation include using recombinant cathepsins in peptide degradation assays [9], studying the effect of cathepsin knock-out mice on the epitope repertoire [10], and using purified endolysosomes from human tissue as a source of proteases for protein degradation assay [11]. In the case of endolysosomal storage disorders, knock-in mice expressing a mutated form of an enzyme have been used to study the role of specific proteins in disease [12].

Each of the above mentioned approaches have some caveats. Endolysosomes comprise a complex set of proteases that are still not completely characterized. Studying the role of a single protease and their contribution to antigen presentation using recombinant proteins is not representative of the complex milieu inside the organelle. Gene knock-out or siRNA studies are helpful in identifying the precise role of a particular protein but do not help in addressing the interactions between the different proteins in the organelle. Though using purified endolysosomes will address the above-mentioned concerns, it involves a laborious, multistep process and results in a much lesser yield. In studies involving limited amount of material such as primary cells from patients, it may not be feasible to purify endolysosomes.

In this study, we describe a simple approach to test the contribution of endolysosomal proteases to protein degradation. Since endolysosomal proteases are active in acidic pH [13], we tested this characteristic by varying the pH in crude peripheral blood mononuclear cell (PBMC) lysate by varying the pH and showed that endolysosomal cathepsin activity can be specifically activated at acidic pH. This simple yet efficient approach enabled us to test the contribution of endolysosomal proteases to several cellular functions using primary cells and therefore is physiologically relevant. We compared these low pH extracts to purified endolysosomes and found that both methodologies produced similar protease activities and peptide degradation kinetics. Here, we exemplify the role of endolysosomes in antigen processing and highlight the potential usefulness of this method to study the function of endolysosomal enzymes in other functions and human diseases.

## Results

### Differential centrifugation of PBMC lysate yields pure endolysosomes

**Figure 1:**
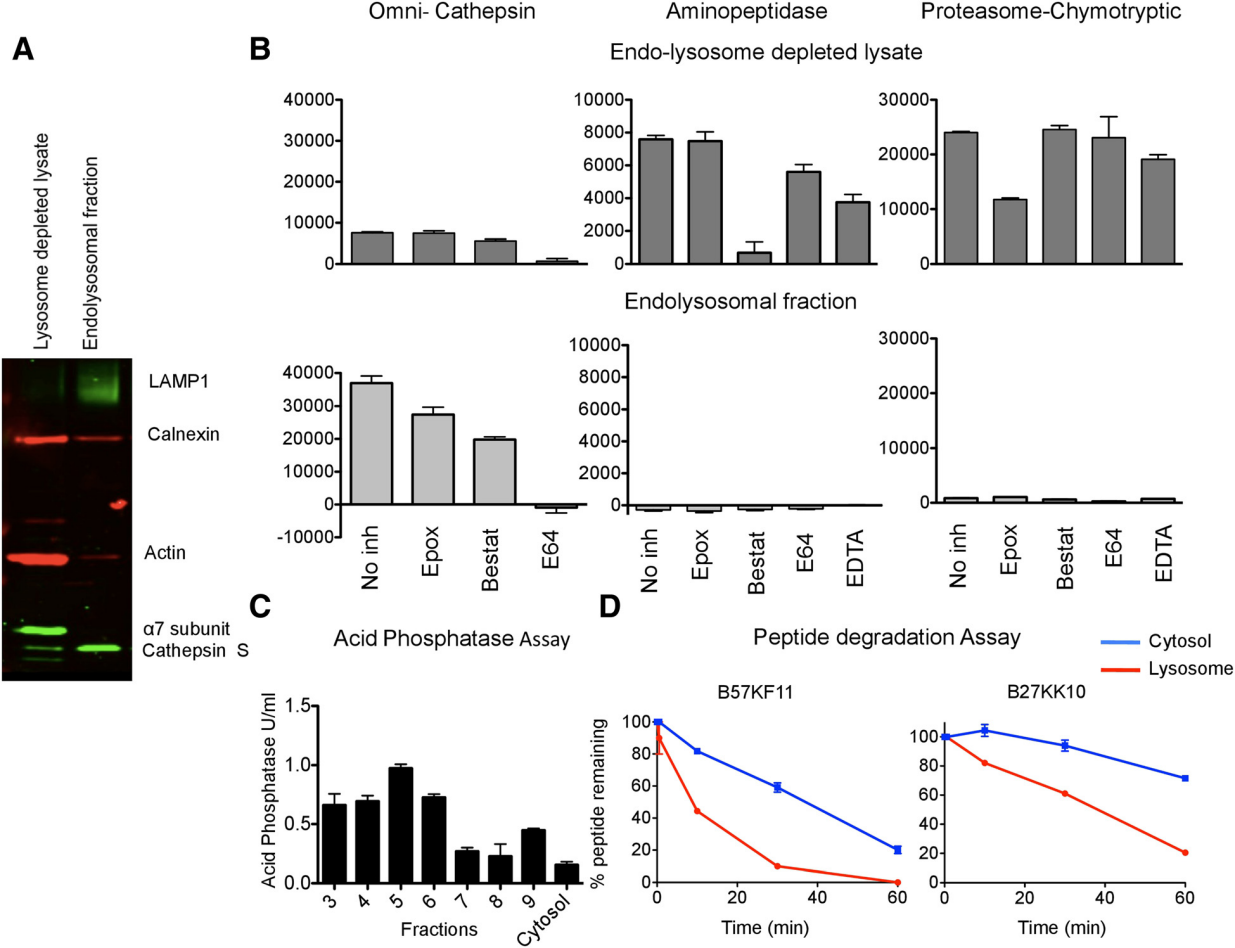
**Differential centrifugation of PBMC lysate yields fractions that are highly enriched in endolysosomes. (A)** Enrichment of endolysosomes in fractions recovered after differential centrifugation was assessed by western blot. PBMC lysate from which endolysosomes were extracted (lane 1), fraction enriched in endolysosomal proteins LAMP1 and Cathepsin S (lane 2). **(B)** Isolated endolysosomal (light grey) and cytosol depleted of lysosomal (dark grey) fractions were assayed for activity of cathepsins, aminopeptidases and chymotryptic activity of proteasome in the presence of inhibitors Epoxomicin (Epox) for proteasome, Bestatin (Best) for aminopeptidase, E64 for cathepsins and EDTA as a metal chelator. Mean and SD of replicates from two different donors are plotted. **(C)** Acid phosphatase assay was performed to confirm enrichment of endolysosomes in different fractions. Figure representative of acid phosphatase activities in one fractionation experiment done in triplicates. Mean and SD are plotted. **(D)** Two HIV Gag peptides, B57KF11 (KAFSPEVIPMF) and B27KK10 (KRWIILGLNK) were subjected to degradation by equivalent amounts of purified cytosol (blue) or endolysosomes (red). An aliquot of the reaction mixture was stopped at periodic intervals and the percentage of original peptide remaining was determined by HPLC. The half-life of the peptide in both conditions was calculated (36 min in cytosol and 8 min in endolysosomes for B57KF11 and more than 60 min in cytosol and 38 min in endolysosome for B27KK10). Figure 1D is representative of two replicates.

We purified endolysosomes from PBMC isolated from the blood of healthy donors. Following differential centrifugation enrichment of lysosomes was confirmed by Western blot by probing for lysosomal associated membrane protein 1 (LAMP1) and cathepsin S. Quantification of the western blot bands showed a six-fold enrichment of cathepsin S in the endolysosomal fraction. This fraction was also devoid of cytosolic protein such as proteasome subunit α7 and actin, and displayed a five-fold reduced expression of endoplasmic reticulum protein, calnexin (Figure 1A). To further confirm the enrichment of endolysosomes, the activities of cathepsin, chymotryptic activity of proteasomes and aminopeptidases were tested using a fluorescence-based activity assay using a fluorogenic peptidase-specific substrate (Suc-LLVY-AMC for chymotryptic activity of proteasomes, H-L-AMC for aminopeptidase and Z-FR-AMC for omnicathepsin) [14-16]. The substrate, made of a short peptide linked to a fluorogenic coumarin derivative fluoresces only when the peptide is cleaved by the enzyme. The level of fluorescence emitted can be quantified over time. Whereas a high level of proteasomal and aminopeptidase activities was detected in the lysosome-depleted cytosolic fraction, purified endolysosomes exhibited high cathepsin activity but no proteasomal activity and aminopeptidase activity (Figure 1B). Specificity of the substrate was measured by monitoring enzyme activity in the presence of specific or irrelevant inhibitors (epoxomicin for proteosome, bestatin for aminopeptidase, EDTA as a metal chelator and E64 for cathepsin). Each activity was strongly inhibited by the inhibitor for the relevant enzyme but not by the other inhibitors. Thirdly, we measured acid phosphatase, an activity enriched in endolysosomes using a colorimetric assay [17]. Acid phosphatase is one of the established lysosomal enzyme markers, and was used to monitor the level of lysosome enrichment in the isolated fractions following differential centrifugation [18]. Comparison of phosphatase activities among different fractions showed that fractions 3 to 6 had the highest phosphatase activity, showing a ten-fold enrichment in acid phosphatase activity compared to the cytosolic fraction (Figure 1C). These three independent approaches confirmed the isolation of endolysosomes and cytosol from crude PBMC lysate.

Our previous studies established that short HIV peptides are variably sensitive to cytosolic degradation, a property that contributes to the efficient presentation of MHC-I epitopes [15]. We reasoned that due to differences in the peptidase content between cytosol (proteasome, aminopeptidases, endopeptidases) and endolysosomes (cathepsins) we may use differential peptide degradation as an additional approach to assess cytosolic and endolysosomal fraction purity, as well as a readout of endolysosomal hydrolytic activities that does not involve fluorogenic substrates. The percentage of acid phosphatase activity in each fraction was used to determine the number of cells that yielded endolysosomes in each fraction. For example, if fraction 4 consisted of 30% of the total acid phosphatase activity, it was assumed that 30% of the initial number of PBMC yielded the endolysosomal proteins in that fraction. Using this information to calculate equivalent amounts of cytosol and endolysosome fractions purified from the same PBMC, we compared the degradation of two HIV p24 Gag-derived epitopes: B27KK10 (KRWIILGLNK, 131-140aa, a ten amino acid long HIV peptide restricted by HLA-B27), and B57KF11 (KAFSPEVIPMF, 30-40aa, an 11-amino acid long HIV peptide restricted by the HLA-B57) [19] (Figure 1D). The disappearance of the peptide was monitored by RP-HPLC, where the amount of peptide is proportional to the surface area under its peak. Upon degradation the peak area reduced and additional peaks corresponding to degradation products appeared [15]. The two HIV peptides were degraded faster in endolysosomes compared to the cytosol. The half-life of B57KF11 was 36 min in the cytosol and decreased to 8 min in endolysosomes. Similarly, the half-life of B27KK10 was greater than 60 min in the cytosol and reduced to 38 min in the endolysosomes, suggesting that the distinct sets of peptidases present in cytosol and endolysosomes differently degraded the two peptides, also providing further evidence of the purity of the PBMC fractions.

### Cathepsin activities can be measured using crude lysate at acidic pH

**Figure 2:**
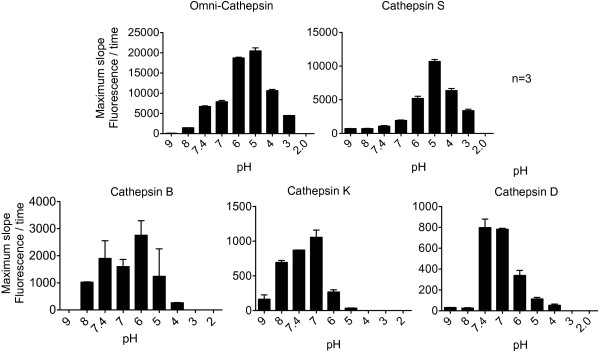
**Optimal pH for activity of cathepsins.** The activities of all cysteine cathepsins (Omni-cathepsin) and cathepsins S, B, K and D present in crude PBMC lysate were assayed using peptidase-specific fluorogenic substrates. The reaction buffer was adjusted so that the pH ranged from 2.0 to 9.0, fluorescence measured over time was plotted and the average maximum slope of the curve from three replicates was determined for each pH.

Purifying endolysosomes from crude lysate is a laborious process and the yield is much lesser. We aimed to develop a simple approach to test the activity of endolysosomal proteins to be used in assays with limited amount of material using crude PBMC lysate. We also made sure that cystatins do not affect cathepsin activity in low pH extracts. Cystatins are cysteine protease inhibitors present in the cytosol and reversibly bind and inhibit the activity of cathepsin [20]. We compared the activity of cysteine cathepsins between live cells and low pH crude lysate derived from the same number of cells. We observed similar levels of enzyme activity in extracts and live cells, showing no effect of cystatins in the measurement of cathepsins in low pH extracts (Additional file 1: Figure S1). Additionally previous studies showed that cystatins are susceptible to protein degradation at low pH [21]. Since the assays we use hereafter are designed to assess cathepsin activity in lower pH, the effect of cystatin activity should be negligible. We first tested the activity of Cathepsin B, K, D, and S present in the endolysosomes at several pH ranging from two to nine, since endolysosomal proteases are activated in acidic pH [22]. Crude PBMC extracts were resuspended in a buffer that was adjusted to the appropriate pH. We then added fluorogenic substrates that were specific for cathepsins S, D, K and B (Z-VVR-AMC for cathepsin S, Ac-RGFFP-AMC for cathepsin D, Z-GPR-AMC for cathepsin K and Z-RR-AMC for cathepsin B) and also an omni-cathepsin substrate (Z-FR-AMC) specific for all cysteine cathepsins [16]. The fluorescence was monitored for three to four hours after addition of crude lysate and substrate. As a negative control, only PBS was added in the place of crude lysate. We then calculated the maximum slope of the fluorescence curve for each cathepsin at all pH after background correction (Figure 2). The experiment was repeated with PBMC from three different healthy donors. Omni-cathepsin substrate was cleaved best at pH 6.0 and 5.0, suggesting that cysteine cathepsin activity is higher at acidic pH. Cathepsin S, which has been implicated in antigen processing showed high activity at pH 5.0 and 4.0. Cathepsin B displayed higher activity at pH 6.0 whereas cathepsin D and K showed higher activity at cytosolic pH 7.4. Several studies attribute endolysosomal enzymes such as Cathepsin S to the production of Class I and II epitopes [6,7,23,24], which exhibited ten-fold higher activity in acidic pH 5.0 when compared to cytosolic pH 7.4. However Cathepsin D and Cathepsin K exhibited eight to ten-fold higher activity in physiological pH 7.4. Recent studies showed that in some cases, cathepsin K is active at normal pH because it is secreted by the cells and play a role in bone resorption [25] and this may explain increased activity of Cathepsin K in cytosolic pH. In summary, PBMC cathepsins specifically involved in protein processing are more active at acidic pH. We also monitored the specificity of each substrate for the enzyme by adding cathepsin inhibitors to the mix and found that cathepsin B substrate can be cleaved by cathepsin S and cathepsin S substrate can be cleaved by cathepsin B (Additional file 2: Figure S2). Despite this cross-reactivity, the overall activity of cathepsin S and cathepsin B covered different pH ranges (4–6 for cathepsin S and 6–8 for Cathepsin B which allows to distinguish them). In addition we used a peptide degradation assay to monitor cathepsin activity at different pH without the use of fluorescent substrates.

### PBMC cathepsins involved in protein processing degrade peptides at acidic pH

**Figure 3:**
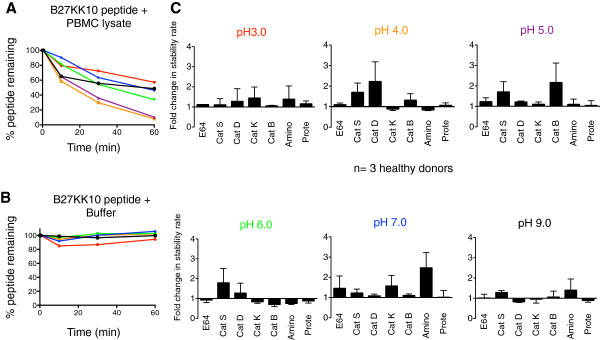
**Stability of B27KK10 peptide in the presence of different inhibitors. (A)** The stability of an HIV peptide, B27KK10 in crude PBMC lysate in various pH buffers was determined using a degradation assay at pH 3.0 (red), pH 4.0 (orange), pH 5.0 (purple), pH 6.0 (green), pH 7.0 (blue), pH 8.0 (black). The amount of remaining peptide was monitored by RP-HPLC during 60 min. **(B)** As a control, the peptide was incubated in the buffer without crude lysate. **(C)** When using inhibitors, the crude lysate was pre-incubated with inhibitors for all cysteine cathepsins (E64), Cathepsin S (Cat S), D (Cat D), K (Cat K), B (Cat B), aminopeptidase (Amino) and proteasome (Prote) for 30min at RT before addition of the peptide. An aliquot of the reaction mixture was stopped at periodic intervals and the percentage of original peptide remaining was determined by HPLC. Based on a one-phase exponential decay, the stability rate of the peptide was calculated. The stability rate of the peptide without any inhibitor was assigned a value of one and the fold change with inhibitors was determined. The average stability rate from three different experiments using PBMC lysate from three different donors was plotted.

To demonstrate a functional consequence of the activation of endolyosomal enzymes at acidic pH we used a peptide degradation assay developed in our laboratory [15] to determine if the cathepsins activated at different pH would degrade peptides differently. These degradation assays were performed in the presence of cathepsin inhibitors to identify specific cathepsins that played a role in antigen processing. B27KK10 was degraded with crude PBMC lysate in a reaction buffer that ranged from pH 3.0 to 9.0. The products of peptide degradation after 0, 10, 30 and 60 minutes of the reaction were analyzed by RP-HPLC and the percentage of remaining original peptide was quantified from the area under peak (Figure 3A). To rule out any effect of acidic pH on the peptides, the peptide was incubated for the same amount of time with the reaction buffer without any crude lysate and the peptide was quantified by RP-HPLC (Figure 3B). As mentioned in Figure 1D, a half-life of the peptide in the presence or absence of cathepsin can be calculated. However, the half-life was not always apparent because of slower degradation kinetics. Therefore, on fitting a one-phase exponential decay curve, we calculated the stability rate of the peptide at each pH (Figure 3C). We previously showed that stability rate correlates with the half-life of a peptide and can be calculated in cases where half-life of the peptide cannot be calculated from the degradation curve [15]. The stability rate of the peptide without inhibitor was assigned a value of one and the fold change in the stability rate in the presence of inhibitors was calculated. In the presence of Cathepsin S and Cathepsin D inhibitors, the stability rate of the peptide increased 1.5- to 3.0-fold at pH 4.0 and 5.0, indicating that the cleavage of the B27KK10 peptide required proteases that were activated only in acidic conditions. Our previous results also showed that cytosolic protease activities are completely eliminated at pH 4.0 and 5.0 [15], therefore indicating that peptide degradation at acidic pH is performed exclusively by endolysosomal proteases. No change in stability rate was noted in extremely acidic pH 3.0 and neutral pH 7.0 and basic pH 8.0. The experiment was repeated using PBMC lysate from three different healthy donors and the average of three results are shown in Figure 3C. In summary, using an alternative approach involving peptide degradation followed by HPLC quantification rather than using fluorogenic enzyme substrates we showed that endolysosomal proteases involved in antigen processing cleave peptides only in acidic pH, and that crude extracts at lower pH can be used to monitor peptide degradation by these cathepsins.

### Endolysosomes and crude lysate at acidic pH degrade peptides with the same kinetics and produce similar fragments

**Figure 4:**
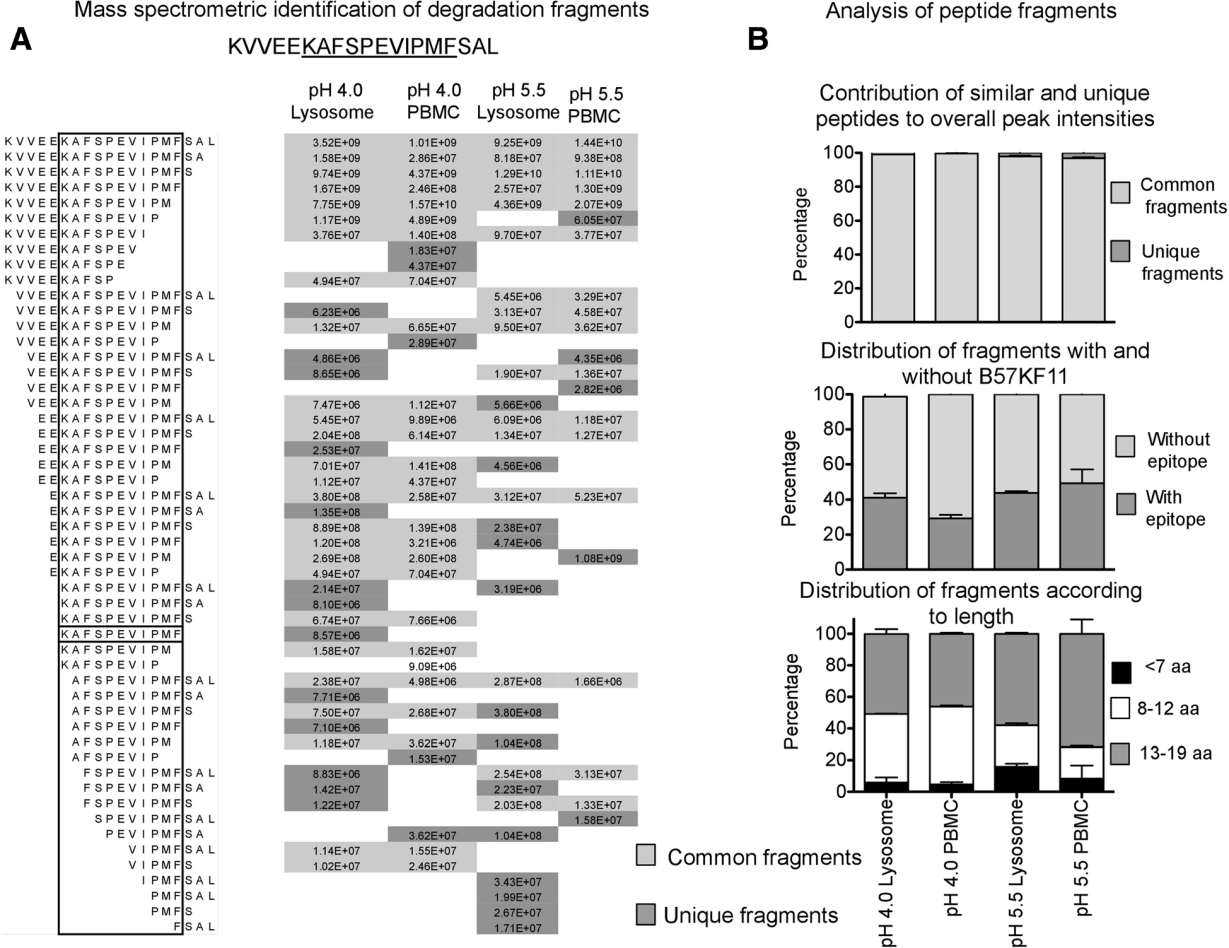
**Degradation of peptides with crude lysate at pH 4.0 is equivalent to using purified endolysosomes. (A)** A long HIV peptide, 5-KF11-3 (KVVEE*KAFSPEVIPMF*AAL) was subjected to degradation using PBMC or purified endolysosomes at pH 4.0 or pH 5.5. The fragments produced were identified by mass spectrometry and the number of similar (light grey) and unique (dark grey) peptides between the two conditions was determined. Peptides that were not detected are indicated in grey letters. The intensity of the peak of each peptide is indicated for each experimental condition. **(B)** The sum of all peptide peak intensities was determined and the distribution of peak intensities in similar (light grey) and unique (dark grey) fragments was calculated (upper). Of the total number of peptide fragments detected in each condition, the percentage of fragments with (dark grey) and without (light grey) the B57KF11 epitope was calculated (middle panel). Similarly, the percentage of fragments that measured 13–19 amino acids in length (dark grey), 8–12 amino acids (white) and lesser than 7 amino acids (black) was determined. In all three cases, the average of the results from two MS runs of the same experiment was plotted.

We then sought to determine if the kinetics of protein degradation and the subsequent peptide fragments produced after degradation were similar when using purified endolysosomes or crude PBMC lysate as a source of enzymes for protein processing. We performed a degradation assay as mentioned in Figure 1 and analyzed the peptide fragments produced by mass spectrometry. We compared the degradation of a longer HIV peptide relevant to antigen processing and peptide-based immunogens with crude lysate and purified endolysosomes at pH 5.5 (late endosomal pH) and 4.0 (lysosomal pH) [26]. Based on results in Figures 2 and 3, we hypothesized that specific sets of cathepsins will be activated at pH 5.5 versus pH 4.0 which would give rise to different peptide fragments between the two conditions. We analyzed the degradation products of a longer peptide, 5-KF11-3 (KVVEE*KAFSPEVIPMF*AAL amino acid residues 25–43), a 19 amino acid long fragment from p24 protein in HIV-1 clade B encompassing epitope B57KF11 Figure (4). The products of the degradation after a 60-minute incubation with either crude lysate or purified endolysosomes at pH 4.0 and 5.5 were identified by mass spectrometry. At pH 4.0, a total of 39 peptides were identified in endolysosome-mediated degradation and 32 peptides in the crude lysate-mediated fraction. Of these, 27 peptides or 84.75% of the fragments present in the crude lysate degradation fraction were similar those produced in endolysosomes (Figure 4A). Unique peptides produced under each condition can be attributed to possible minor contribution cytosolic proteases that retain some hydrolytic activity in low pH. At pH 5.5 we found that 76.2% of the peptides present in crude lysate mediated degradation fraction were similar to the endolysosomal fraction. Interestingly, common peptide fragments detected in crude lysate and endolysosomal degradation conditions contributed to 99% of overall peptide intensity showing that unique fragments detected were present only in negligible quantities (Figure 4B, upper panel). The peptide fragments produced in both degradation conditions at each pH ranged from 4–19 amino acid residues in length with several fragments harboring the MHC class I epitope B57KF11. Fragments produced at pH 5.5 in crude lysate and endolysosomes were mostly longer with 70% of the fragments between 13–19 amino acids in length and fragments produced at pH 4.0 were shorter, suggesting a stepwise degradation of longer fragments into shorter peptides over the course of endolysosomal maturation (Figure 4B, lower panel). Also, on average 40% of the peptides produced in pH 5.5 in endolysosomes and crude lysate and 30% of the peptides in pH 4.0 contained extensions of the epitope B57KF11, showing that similar levels of peptide degradation were present in both conditions (Figure 4B, middle panel). We repeated the degradation using lysate from a different PBMC donor and the peptide 5-ISW9-3 (MVHQA*ISPRTLNAW*VKV), a 17 amino acid long peptide from the HIV p24 protein encompassing epitope B57ISW9 (Additional file 3: Figure S3). We compared by mass spectrometry the identity and corresponding peak sirface intensities of all peptides produced in purified endolysosomes and in crude lysate at pH 4 and 5.5 (Additional file 3: Figure S3A). More than 95% of the peptide intensities were contributed by fragments that were similar between PBMC crude lysate and endolysosomes (Additional file 3: Figure S3B, upper panel). Comparable levels of peptides encompassed the epitope B57ISW9 and peptide length distributions were observed between crude lysate and endolysosomes (Additional file 3: Figure S3B, middle and lower panels). However, in contrast to 5-KF11-3 the peptides generated at pH 4.0 and pH 5.5 were similar for 5-ISW9-3, suggesting that the peptides were generated by cathepsins active at pH 4 and 5.5. The differences in the degradation patterns of 5-KF11-3 and 5-ISW9-3 showed that peptide fragments generated by cathepsin-mediated degradation is dependent on the nature of the oligopeptide and its susceptibility to cleavage by cathepsins present in different compartments. We also determined the half-life of B57KF11 peptide in crude extracts at low pH versus endolysosomes and found them to be similar (8 min in endolysosomes and 6 min in crude lysate, both at pH 4.0) (data not shown). These results in summary show that crude lysate at pH 4.0 to pH 5.5 can be used to assess cathepsin-mediated degradation of peptides and proteins in the place of purified endolysosomes.

## Discussion

Endolysosomes contain proteins that are involved in diverse functions in the cell [1]. Loss of activity in several of these proteins leads to disease [2-5]. Current approaches to study endolysosomal proteins are not sufficient to comprehensively understand the role of the organelle in cellular functions and using purified organelles is not feasible in studies involving access to limited amount of material such as primary cells and tissues. Because several endolysosomal proteases are activated in acidic pH [13], we have utilized this characteristic to develop a simple approach to study the role of these proteases in peptide degradation using crude PBMC lysate.

Most studies involving endolysosomal protease activity use recombinant purified cathepsins [9], manipulations such as gene knockouts in *in vitro* cell cultures [10] or purified endolysosomes from cells or tissues [11]. In some cases, the results obtained are not the most physiologically relevant and require further validation in primary human tissues. Using the methodology described here, it is possible to assess the activity of one or simultaneously, many endolysosomal enzymes using crude lysate prepared from primary cells. Also, by modulating the pH, specific enzymes can be activated and cross-reactivity from other cytosolic proteins can be avoided while allowing to follow protein degradation in endolysosomal compartments. By using appropriate substrates for each enzyme a novel diagnostic for lysosomal protein disorders may be envisioned.

Another area of research that will benefit greatly from this assay is antigen presentation. Antigen presentation takes place in two different pathways inside the cell; the endogenous cytosolic pathway predominantly involved in class I presentation and the exogenous endolysosomal pathway involved in class II and class I epitope cross-presentation [27]. One of the questions that has yet to be addressed in antigen presentation is the characteristics that govern efficient presentation of epitopes from a pathogen-derived protein endocytosed in cells. To date, the precise nature by which long pathogen-derived proteins are degraded in endocytic compartments to produce epitope length fragments is poorly defined. Despite the critical role of protein degradation in endolysosomes to elicit immune responses the nature of peptides cross-presented by MHC-I and MHC-II is not well defined in part due to the lack of adequate assays. Class II epitopes are not as well characterized as class I epitopes because of the limited knowledge of anchor residues within the MHC class II protein and looser binding of peptides onto MHC-II. Using our assay, it will be possible to study the degradation of pathogen-derived proteins or vaccine immunogens in crude extracts at neutral vs. acidic pH and identify epitopes produced in cytosol and endolysosomal compartments by mass spectrometry. Using these peptide fragments in *in vivo* antigen presentation assays and assaying for CD4 or CD8 T cell stimulation will further help to define class I and class II epitopes that are produced after intracellular processing and cross-presentation.

Vaccine immunogens (inactivated pathogens, purified proteins, peptides, viral vectors) mostly enter dendritic cells through endolysosomes for degradation followed by presentation of peptides by MHC-I and MHC-II leading to the priming of CD8 T cell responses and CD4 T helper cell responses [27]. Knowledge of epitopes that are well defined and preserved within cellular compartments will enable efficient vaccine design. This is especially important in light of chimeric vaccine immunogens (which includes vaccines made of protein fragments separated by linkers, peptides derived from conserved regions of proteins that do not undergo mutations or peptides that include both wild type and mutated versions separated by linkers), so as to include peptides that will be efficiently processed and presented on the cell surface [28]. With increased interest in targeted vaccines, wherein, antigens can be engineered to target specific compartments, once again, knowledge of stable epitopes that are produced efficiently in each of the targeted compartments is of immense importance. The methodology described here will facilitate these studies and bypass the need of intensive labor, access to large amounts of primary human cells and complicated techniques.

## Conclusions

In summary, we have shown that using crude PBMC cellular extracts at low pH, endolysosomal protease activity can be measured in a simple and efficient manner, results of which can be used in several applications. We demonstrated that by varying the pH in crude cellular extracts, we could specifically activate certain cathepsins at each pH and thereby monitor protease activity over the course of endolysosomal maturation. We demonstrated the usefulness of this assay in the context of antigen processing and showed comparable results to using purified endolysosomes.

## Methods

### Human studies

Buffy coats from blood donated by anonymous healthy donors were purchased from Massachusetts General Hospital. Partners Human Research Committee (Boston, MA) approved the use of buffy coats under protocol no. 2005P001218.

### Reagents and antibodies

Antibodies for Lysosome Associated Membrane Protein 1 (LAMP1) (sc-20011) and cathepsin S (sc-271619) were purchased from Santa Cruz, beta actin (ab8227) and calnexin (ab22595) from Abcam and proteasome α 7 subunit (BML-PW8110-0100) from Enzo Lifesciences. All chemicals were purchased from Sigma and fluorogenic substrates were purchased from Enzo lifesciences. Highly purified HIV peptides were purchased from Massachusetts General Hospital peptide core facility or from Biosynthesis.

### Endolysosome purification

Endolysosomes were purified from crude PBMC lysate by differential centrifugation. PBMCs were isolated from human buffy coats using Ficoll centrifugation [29]. Cells resuspended in extraction buffer at a concentration of 5 × 10^8^ cells/ml [30] were lysed using glass beads as in a previous study done in our laboratory [31]. The cell lysate was centrifuged at 1000g for 10 min at 4°C. The supernatant was transferred to a new tube and centrifuged at 20,000 g for 20 min at 4°C. The resulting pellet consists of endoplasmic reticulum, mitochondria and endolysosomes. The supernatant corresponds to purified cytosolic fraction. A step gradient ranging from 23% to 8% was built using an Optiprep based endolysosome isolation kit (Sigma, LYSISO1). The tubes were centrifuged at 100,000 g in a Beckmann Coulter tabletop centrifuge at 4°C. 120 μl fractions were collected and further analyzed for endolysosomal enrichment.

### Western blot

Following endolysosomal purification, 10μl of each fraction and the post nuclear supernatant were loaded on a 4-12% SDS PAGE gradient gel (Invitrogen) run at a constant 185V. The proteins were transferred to a PVDF membrane (Fisher Scientific) using a semi-dry blotting system (Bio-Rad) for 35 min at 20V. The membrane was blocked using a blocking buffer [30] for 1 hour at RT followed by incubation with the primary antibodies overnight at 4C. The membrane was washed three times, 10 min each with 0.1% TBS Tween followed by incubation with secondary antibodies, Goat anti rabbit (Licor, 926–68071) and Donkey anti mouse (Licor, 926–32212) for 1 hour at RT. The membrane was washed again for three times in wash buffer and imaged using a Licor imaging system.

### Enzyme activity assay

Activities of proteases were tested using a fluorogenic enzyme substrate specific for each endolysosomal cathepsin [16]. For endolysosomal purification samples, 3 μl of each fraction was added to 96 well plates. For analyzing cathepsin activity in different pH, 3 μg of crude PBMC lysate was added to each well. Appropriate amount of the enzyme specific substrate (omni-cathepsin 50 μM, chymotryptic activity of proteasome 100 μM and aminopeptidase 12.5 μM, cathepsin S 50 μM, cathepsin L 50 μM, cathepsin D 10 μM, cathepsin K 50 μM, cathepsin B 50 μM) resuspended in 200 μl of buffer (20 mM HEPES, 50 mM potassium acetate, 5 mM magnesium chloride, 1 mM ATP, 1mM DTT for proteasome, same buffer without ATP and DTT for aminopeptidase and 50 mM sodium chloride, 50 mM potassium phosphate, 2 mM EDTA and 2 mM DTT for cathepsins) were added to each well containing extracts or buffer (to control for background fluorescence). Fluorescence was measured every 5 minutes for 3 hours using a Victor-3 plate fluorescence reader (Perkin Elmer, Boston, MA) as described before [29]. The resulting fluorescence curves were plotted over time. For each condition, the maximum rate of fluorescence emission was calculated after background correction, which is equivalent to the maximal rate of proteolytic hydrolysis. To verify the specificity of each substrate hydrolysis, crude lysate was pre-incubated with appropriate amounts of peptidase inhibitors: ZFL-COCHO 10 μM to inhibit Cathepsin S, pepstatin 100μM to inhibit Cathepsin D, BML-244 10 μM to inhibit Cathepsin K, E64 50 μM to inhibit all cysteine cathepsins, MG 132 10 μM to inhibit proteasome and bestatin 12.5 μM to inhibit aminopeptidase for 30 minutes at room temperature before addition of the fluorogenic substrate. Maximum slopes of the fluorescence over time curves in the presence or absence of inhibitors showed a 35-100% specific inhibition of each activity (data not shown).

### Acid phosphatase assay

The presence of acid phosphatase in endolysosomal fractions was confirmed using a kit from Sigma (CS0740). 10 μl of each fraction was incubated with the substrate in the provided substrate buffer for 10 min at 37°C. 200 μl of the stop solution (2M sodium hydroxide) was added and the absorbance at 405 nM was measured. A blank without any lysate serving as a negative control and pure enzyme serving as a positive control was also included in the experiment. The amount of acid phosphatase present was calculated using the formula, Units/ml = (A_405_(sample)-A_405_(blank)) × 0.05 × 0.3 × DF/A_405_(standard)x time x V_enz,_ where DF is dilution factor and V_enz_ is volume of the pure enzyme.

### Peptide degradation assay

The assay is adapted from previously published studies from our laboratory [31]. 8 nM of peptide was incubated with 30 μg of crude PBMC lysate in 250 μl of degradation buffer (20 mM HEPES, 1 mM magnesium chloride, 137 mM potassium acetate, 1 mM ATP) that was adjusted to the appropriate pH. In studies involving purified endolysosome, an acid phosphatase assay was performed on the fractions and the amount of acid phosphatase activity was used to calculate the total number of cells from which each fraction was derived. Using this approach, equivalent amount of cytosol and endolysosome that were derived from the same number of cells were used in peptide degradation assays. For the mass spectrometry analysis of peptide fragments, the level of omni-cathepsin enzyme activity was assessed using the activity assay and volume of crude lysate and purified endolysosomes containing similar levels of the enzyme activity was used in the degradation assay. When using inhibitors, the compound was added to the crude lysate before addition of the peptide and incubated at room temperature for 30 minutes. The mixture was incubated at 37°C and at each time point 50 μl were collected and the reaction was stopped by adding 2.5 μl of 100% trifluoroacetic acid. Another 50 μl of the degradation buffer was added to each sample and the amount of peptide remaining was analyzed by RP-HPLC.

### Mass spectrometry analysis of peptide fragments

Following peptide degradation, the peptide fragments produced were enriched using 10% trichloroacetic acid precipitation and identified by in-house mass spectrometry analysis as previously described [14]. After diluting the product to 500 femtomole in 80% water, 15% acetonitrile, 5% trifluoroethanol, the peptides were resolved in RP-HPLC on a C18 column (Nano-LC Eksigent) and electrosprayed on an Orbitrap discovery mass spectrometer (Thermo). The peptides were identified using Proteome Discoverer software (Thermo) and the surface intensity of each peptide peak corresponded to the amount of peptide present. The peptide fragments were aligned using the Jalview software and number of fragments present in both the conditions were used to calculate the percentage of similar peptides detected. The experiments were repeated twice using two different endolysosomal preparations and degradation products of each experiment were analyzed twice with the mass spectrometer.

## Abbreviations

PBMC: Peripheral blood mononuclear cells; MHC: Major histocompatibility complex; LAMP1: Lysosomal associated membrane protein 1; HIV: Human immunodeficiency virus; RP-HPLC: Reversed phase high performance liquid chromatography.

## Competing interests

The authors declare that they have no competing interests.

## Authors’ contributions

AV and SLG conceived the study, designed and planned experiments and wrote the manuscript. AV carried out the lysosome purification, degradation and activity assays. ED performed all the western blots. NYL performed activity assays. JB, YX and MS defined the conditions and ran the samples in a mass spectrometer. MG performed the acid phosphatase assays. All authors read and approved the manuscript.

## Supplementary Material

Additional file 1: Figure S1 Cysteine cathepsin activity is similar in live cells and lysate. Human primary CD4 T cells were grown in R10-IL2. 5 × 10^4^ cells resuspended in PBS was added to each well. For lysate, the volume of the lysate derived from 5 × 10^4^ cells was calculated and added to each well. When using inhibitors, E64 was added to the well and preincubated for 30 min at 37C. Following incubation, the activity of an omnicathepsin substrate was measured. Average and SD of 3 experiments. Click here for file

Additional file 2: Figure S2 Cathepsin S and B substrates are cleaved by both enzymes. Cathepsin B and S activity was measured in crude PBMC lysate resuspended in pH 4.0 using fluoregenic subtrates. When using inhibitors, lysate was preincubated with Cathepsin S, D, K, B and an omnicathepsin inhibitor following which activity of the enzyme was measured. The average maximum slope of the curve derived from three replicates quantified in relative fluorescent units (RFU)/min is plotted. Click here for file

Additional file 3: Figure S3 Degradation of peptides with crude lysate at pH 4.0 is equivalent to using purified endolysosomes for the oligopeptide B57-5ISW9-3. A) A long HIV peptide, 5-ISW9-3 (MVHQA*ISPRTLNAW*VKV) was subjected to degradation using PBMC or purified endolysosomes at pH 4.0 or pH 5.5. The fragments produced were identified by mass spectrometry and the number of similar (light grey) and unique (dark grey) peptides between the two conditions was determined. Peptides that were not detected are indicated in grey letters. The surface of the peak of each peptide is indicated for each experimental condition. (B) The sum of all peptide peak intensities was determined and the distribution of peak intensities in similar (light grey) and unique (dark grey) fragments was calculated (upper). Of the total number of peptide fragments detected in each condition, the percentage of fragments with (dark grey) and without (light grey) the B57ISW9 epitope was calculated (middle panel). Similarly, the percentage of fragments that measured 13–19 amino acids in length (dark grey), 8–12 amino acids (white) and lesser than 7 amino acids (black) was determined. In all three cases, the average of the results from two MS runs of the same experiment was plotted. Click here for file
