# Supplementary figures and images for: A simple methodology to assess endolysosomal protease activity involved in antigen processing in human primary cells
Source: BMC Cell Biol. 2013 Aug 9;14:35. doi: 10.1186/1471-2121-14-35 (PMC3751085; doi:10.1186/1471-2121-14-35)

## Supplementary Figure 1

Comparison of Omnicathepsin activity between live cells and lysate

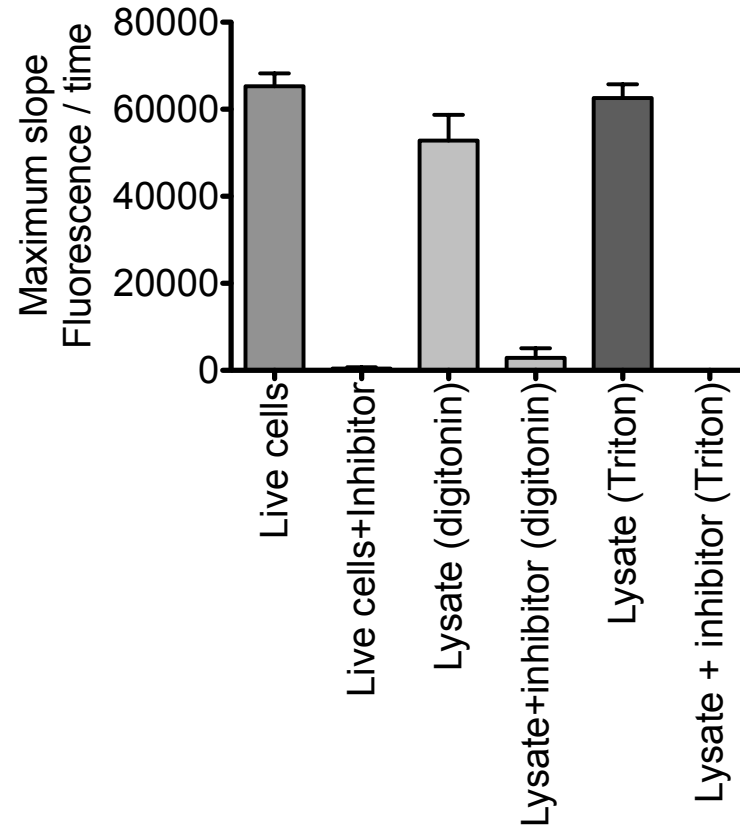

Supplement: Additional file 1: Figure S1 — Cysteine cathepsin activity is similar in live cells and lysate. Human primary CD4 T cells were grown in R10-IL2. 5 × 10^4^ cells resuspended in PBS was added to each well. For lysate, the volume of the lysate derived from 5 × 10^4^ cells was calculated and added to each well. When using inhibitors, E64 was added to the well and preincubated for 30 min at 37C. Following incubation, the activity of an omnicathepsin substrate was measured. Average and SD of 3 experiments. [file 1471-2121-14-35-S1.pdf]

Supplementary Figure 2

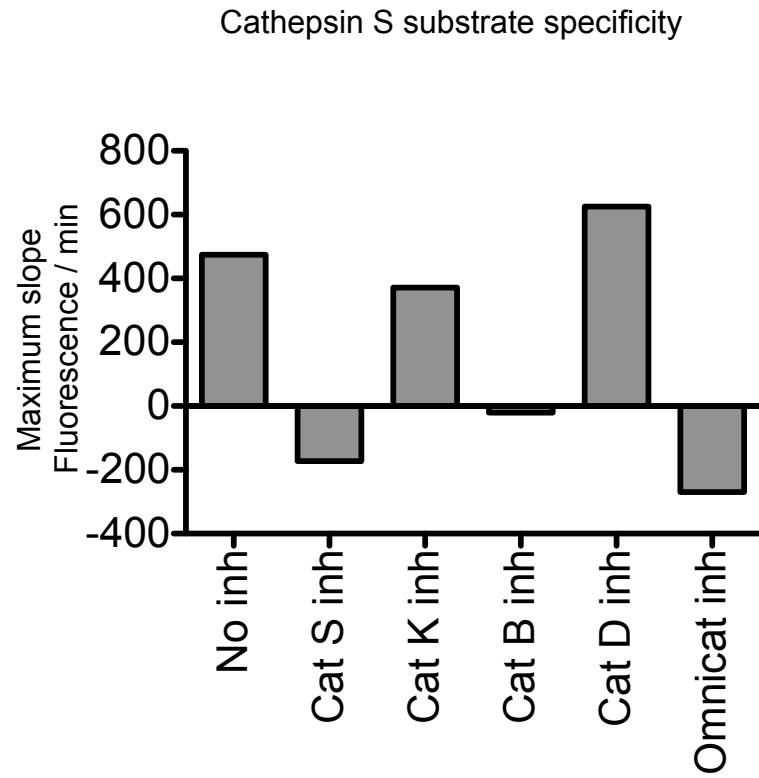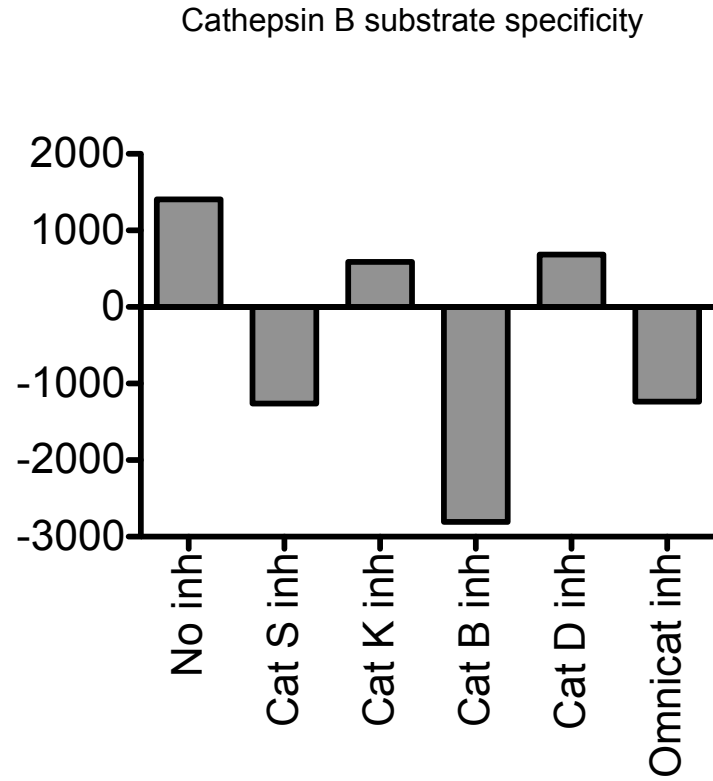

Supplement: Additional file 2: Figure S2 — Cathepsin S and B substrates are cleaved by both enzymes. Cathepsin B and S activity was measured in crude PBMC lysate resuspended in pH 4.0 using fluoregenic subtrates. When using inhibitors, lysate was preincubated with Cathepsin S, D, K, B and an omnicathepsin inhibitor following which activity of the enzyme was measured. The average maximum slope of the curve derived from three replicates quantified in relative fluorescent units (RFU)/min is plotted. [file 1471-2121-14-35-S2.pdf]
