# Supplementary material for: A simple methodology to assess endolysosomal protease activity involved in antigen processing in human primary cells
Source: BMC Cell Biol. 2013 Aug 9;14:35. doi: 10.1186/1471-2121-14-35 (PMC3751085; doi:10.1186/1471-2121-14-35)

Supplementary Figure 3

A

MVHQA<u>ISPRTLNAW</u>VKV

Peptide	pH 4.0 Lysosome	pH 4.0 PBMC	pH 5.5 Lysosome	pH 5.5 PBMC
MVHQAISPRTLNAWVKV	1.05E+10	2.36E+09	8.62E+09	4.01E+09
MVHQAISPRTLNAWVK	7.14E+08	1.50E+09	7.68E+08	3.57E+09
MVHQAISPRTLNAWV	4.25E+08	4.73E+08	3.36E+08	4.35E+08
MVHQAISPRTLNAW	1.04E+09	2.60E+09	8.24E+08	6.74E+08
MVHQAISPRTLNA	6.32E+08	2.98E+09	6.04E+08	1.00E+09
MVHQAISPRTLN	1.16E+08	2.50E+08	1.62E+08	2.53E+08
MVHQAISPRTL	2.64E+08	2.98E+08	4.37E+08	4.05E+08
MVHQAISPRT	2.04E+07	2.71E+07	2.59E+07	2.56E+07
MVHQAISPR	3.34E+07	4.16E+07	4.43E+07	3.80E+07
MVHQAIS	7.06E+07	3.91E+07	4.25E+07	2.41E+07
VHQAISPRTLNAWVKV	2.20E+08	8.13E+07	3.07E+08	2.33E+08
VHQAISPRTLNAWVK	1.22E+08	4.72E+07	2.26E+07	3.51E+08
VHQAISPRTLNAWV		2.11E+07	1.39E+07	6.59E+07
VHQAISPRTLNAW	2.13E+07	6.13E+07	3.53E+07	1.68E+08
VHQAISPRTLNA	3.11E+06	8.43E+07	1.23E+07	9.68E+07
VHQAISPRTLN	1.80E+06	6.35E+06	3.38E+06	1.83E+07
VHQAISPRTL	3.53E+06	5.34E+06	1.11E+07	2.09E+07
VHQAISPR	6.93E+06	7.40E+06	7.73E+06	6.77E+06
HQAISPRTLNAWVKV	1.22E+08	9.67E+07	1.19E+08	3.18E+08
HQAISPRTLNAWVK	6.66E+06	8.46E+07	1.55E+07	2.47E+08
HQAISPRTLNAWV		8.15E+06		1.31E+07
HQAISPRTLNAW		4.27E+07		2.10E+07
HQAISPRTLNA	1.66E+06	5.85E+07		3.13E+07
HQAISPRTLN		4.06E+06		5.76E+06
HQAISPRTL		8.47E+06	2.80E+06	1.56E+07
HQAISPR	2.50E+06	2.52E+06	2.62E+06	2.51E+06
QAISPRTLNAWVKV	5.48E+07	1.90E+07	5.33E+07	5.94E+07
QAISPRTLNAWVK		1.01E+07		2.67E+07
QAISPRTLNAWV				6.29E+06
QAISPRTLNAW		1.13E+07		1.37E+07
QAISPRTLNA		6.05E+06		
QAISPRTLN				8.33E+05
QAISPRT		4.19E+06		
AISPRTLNAWVKV	1.48E+07	4.91E+07	1.07E+07	9.97E+07
AISPRTLNAWVK		2.22E+07		8.77E+07
AISPRTLNAWV		1.23E+07		1.81E+07
AISPRTLNAW		3.24E+07		3.46E+07
AISPRTLNA	1.83E+06	3.19E+07		1.80E+07
AISPRTLN	4.65E+07	1.19E+07	3.48E+07	1.79E+07
AISPRTL		1.86E+06		3.07E+06
ISPRTLNAWVKV	3.60E+07	1.06E+08	3.56E+07	8.63E+07
ISPRTLNAWVK		3.11E+07		7.08E+07
ISPRTLNAWV		2.27E+07		8.76E+06
ISPRTLNAW		6.99E+07	2.13E+06	2.05E+07
ISPRTLNA	1.29E+06	5.83E+07	8.27E+05	1.13E+07
ISPRTLN		4.05E+06		1.74E+06
ISPR	1.40E+06	1.45E+06		1.42E+06
SPRTLNAWVKV	5.98E+07	2.77E+07	6.53E+07	3.37E+07
SPRTLNAWVK	1.48E+06	5.77E+06	7.37E+06	5.60E+07
SPRTLNAWV				6.52E+06
SPRTLNAW		2.22E+06		
PRTLNAWVKV	3.71E+07	1.72E+07	2.73E+07	1.08E+07
PRTLNAWV		1.82E+06		
RTLNAWVKV	1.06E+07		9.88E+06	
RTLNAW				1.11E+06
TLNAWVKV	9.65E+07	5.91E+07	8.94E+07	7.27E+07
TLNAWVK	2.50E+06	9.73E+06	4.68E+06	3.79E+07
TLNAWV				2.69E+06
LNAWVKV	1.62E+07	7.88E+06	1.98E+07	6.06E+06
NAWVKV			2.72E+06	2.43E+06
AWVKV	2.31E+06	2.93E+06	2.95E+06	2.03E+06
WVKV	2.47E+07	1.69E+08	2.80E+06	5.14E+07

Common fragments

Unique fragments

B

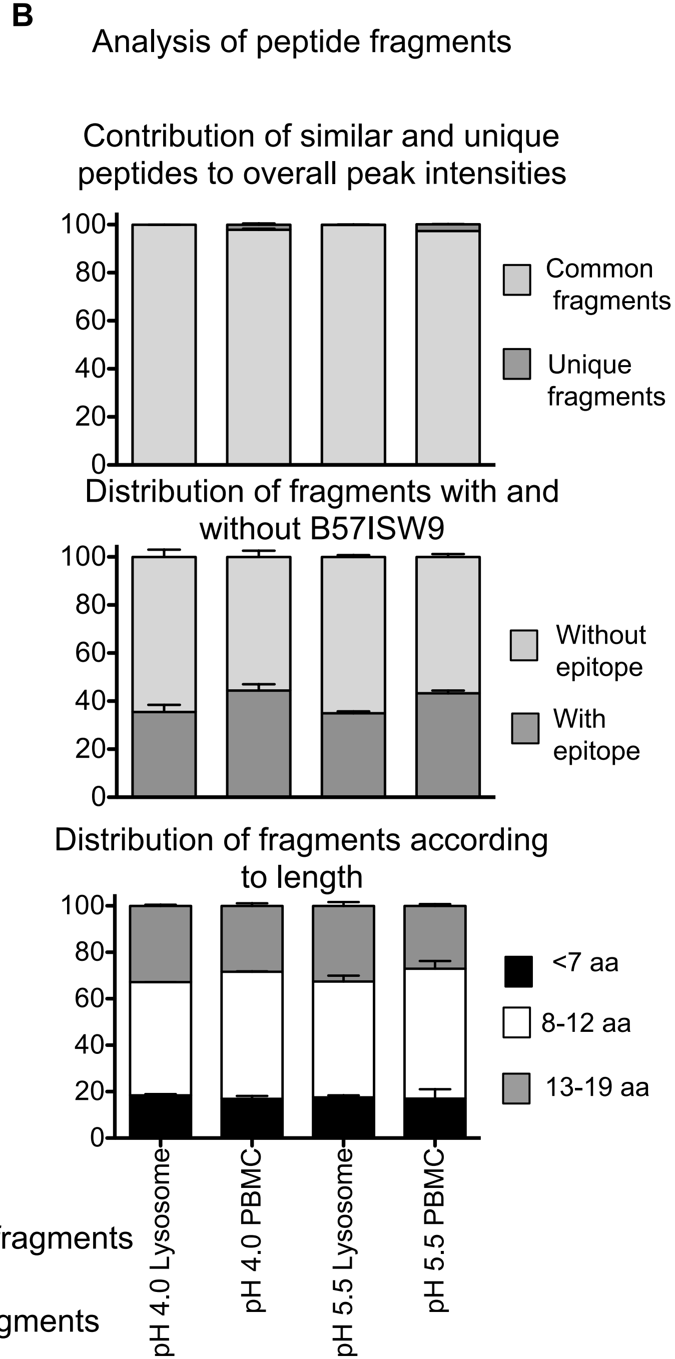

Supplement: Additional file 3: Figure S3 — Degradation of peptides with crude lysate at pH 4.0 is equivalent to using purified endolysosomes for the oligopeptide B57-5ISW9-3. A) A long HIV peptide, 5-ISW9-3 (MVHQAISPRTLNAWVKV) was subjected to degradation using PBMC or purified endolysosomes at pH 4.0 or pH 5.5. The fragments produced were identified by mass spectrometry and the number of similar (light grey) and unique (dark grey) peptides between the two conditions was determined. Peptides that were not detected are indicated in grey letters. The surface of the peak of each peptide is indicated for each experimental condition. (B) The sum of all peptide peak intensities was determined and the distribution of peak intensities in similar (light grey) and unique (dark grey) fragments was calculated (upper). Of the total number of peptide fragments detected in each condition, the percentage of fragments with (dark grey) and without (light grey) the B57ISW9 epitope was calculated (middle panel). Similarly, the percentage of fragments that measured 13–19 amino acids in length (dark grey), 8–12 amino acids (white) and lesser than 7 amino acids (black) was determined. In all three cases, the average of the results from two MS runs of the same experiment was plotted. [file 1471-2121-14-35-S3.pdf]
